# A mixed-methods analysis to understand the implementation of a multistakeholder research consortium: Environmental influences on child health outcomes (ECHO)

**DOI:** 10.1017/cts.2023.620

**Published:** 2023-08-29

**Authors:** Elissa Z. Faro, Katherine A. Sauder, Gwendolyn S. Norman, Amber Anderson, Carmen Vélez-Vega, David Napp, Kathi C. Huddleston

**Affiliations:** 1 Department of Internal Medicine, Carver College of Medicine, University of Iowa, Iowa City, IA, USA; 2 Department of Pediatrics, Section of Nutrition, University of Colorado School of Medicine, Aurora, CO, USA; 3 Department of Obstetrics and Gynecology, Wayne State University, Detroit, MI, USA; 4 Duke Clinical Research Institute, Duke University, Durham, NC, USA; 5 Social Sciences Department, Graduate School of Public Health, University of Puerto Rico, San Juan, Puerto Rico; 6 Practical Applications of Public Health, Durham, NC, USA; 7 College of Public Health, George Mason University, Fairfax, VA, USA

**Keywords:** Team science, implementation science, stakeholder engagement, qualitative, ethnography

## Abstract

**Introduction:**

Large, transdisciplinary research consortia have increasingly been called upon to address complex and challenging health problems. The National Institutes of Health’s (NIH) Environmental influences on Child Health Outcomes (ECHO) Program developed multisite collaboration strategies to promote impactful collaborative observational research on child health. Team science and implementation science offer theoretical and methodological structure to answer questions about the strategies that facilitate successful consortia. We sought to characterize the elements and conditions that influence the implementation of a complex, interdisciplinary longitudinal research program, ECHO.

**Methods:**

Informed by the Practical, Robust, Implementation and Sustainability Model, our ethnographic research included semi-structured interviews with internal stakeholders and program evaluation metrics. We conducted template and matrix analysis and triangulated the qualitative and quantitative data to understand the implementation of ECHO.

**Results:**

Between February and May 2022, we conducted 24 virtual interviews with representatives from ECHO components. The main cross-cutting topics that emerged from thematic analysis were collaboration and team science; communication and decision-making; data processes and harmonization; and diversity, equity, and inclusion. Both the qualitative and secondary quantitative evaluation data provided insights into the reach, adoption, implementation, and effectiveness of the program.

**Conclusion:**

A large, multidisciplinary research consortium such as ECHO has produced conceptual, instrumental, capacity building, and connectivity impact for internal and external stakeholders. Facilitators included infrastructure that supported collaboration and learning, alignment of data processes, and harmonization. Opportunities for enhanced impact include multidisciplinary, multimethod communication strategies, and alignment of research priorities.

## Introduction

Large research collaboratives often have more success than single investigators conducting research alone; they produce more publications in journals with higher impact factors and are cited more frequently [1,2]. This can lead to higher impact on programs and policies as this research reaches larger stakeholder audiences. As a result, funding agencies are increasing their support for large, transdisciplinary research consortia to address complex, challenging health problems [3]. There has been an accompanying rise of team science research to understand strategies that facilitate successful teams (e.g., communication, leadership) [4]. As part of the National Institutes of Health’s (NIH) focus on multiple cross-disciplinary programs and research centers [5,6], the Environmental influences on Child Health Outcomes (ECHO) Program developed a multisite collaboration to promote impactful, collaborative research [7].

**Figure 1.**
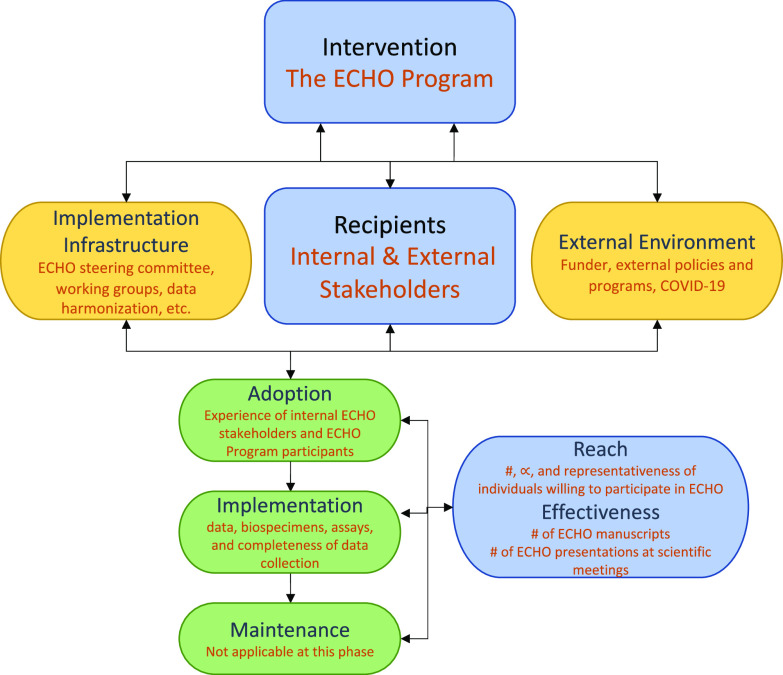
The PRISM framework [12] with details specific to our ECHO program research.

Implementation science offers a structure to understand the identifiable contextual factors that impact implementation of the ECHO Program. These factors, such as policies, organizational climate, incentives, workflow, and target population, are multilevel and complex, and related to implementation outcomes [8,9]. Despite the increasing calls for team science, there are still a number of outstanding questions to be addressed, such as the effects of research structures and funding mechanisms on team functioning [4,10]. We sought to develop an in-depth understanding of the implementation of the observational ECHO Program and compare qualitatively assessed contextual factors to quantitative implementation outcomes. The Practical, Robust Implementation and Sustainability Model (PRISM) guided our research design, data collection, analysis, and the integration of findings to better understand the contextual factors that impact the implementation of a multistakeholder consortium [11]. We chose PRISM as our framework to provide the structure for understanding the complex system of ECHO and how its components interact. PRISM has four major domains: (1) intervention (the ECHO Program); (2) recipients (program internal and external stakeholders); (3) implementation and sustainability infrastructure (ECHO steering committee, working groups, data harmonization, etc.); and (4) external environment. The external environment comprised the funder, external stakeholders’ policies, and programs, and, ultimately, the COVID-19 pandemic. We captured implementation outcomes qualitatively and quantitatively using the Reach Effectiveness Adoption Implementation and Maintenance (RE-AIM) framework (Fig. 1) [8,12–14].

We focused on characterizing the context for the implementation of the ECHO Program by using ethnographic methods; triangulating the qualitative interview data with quantitative implementation outcomes, a preexisting subset of the measures developed by ECHO to track progress toward program goals [8,14]. We present our qualitative and quantitative findings to understand how internal stakeholders perceive the facilitators and barriers to successful implementation of a large research consortium.

## Materials and Methods

### Setting

**Figure 2.**
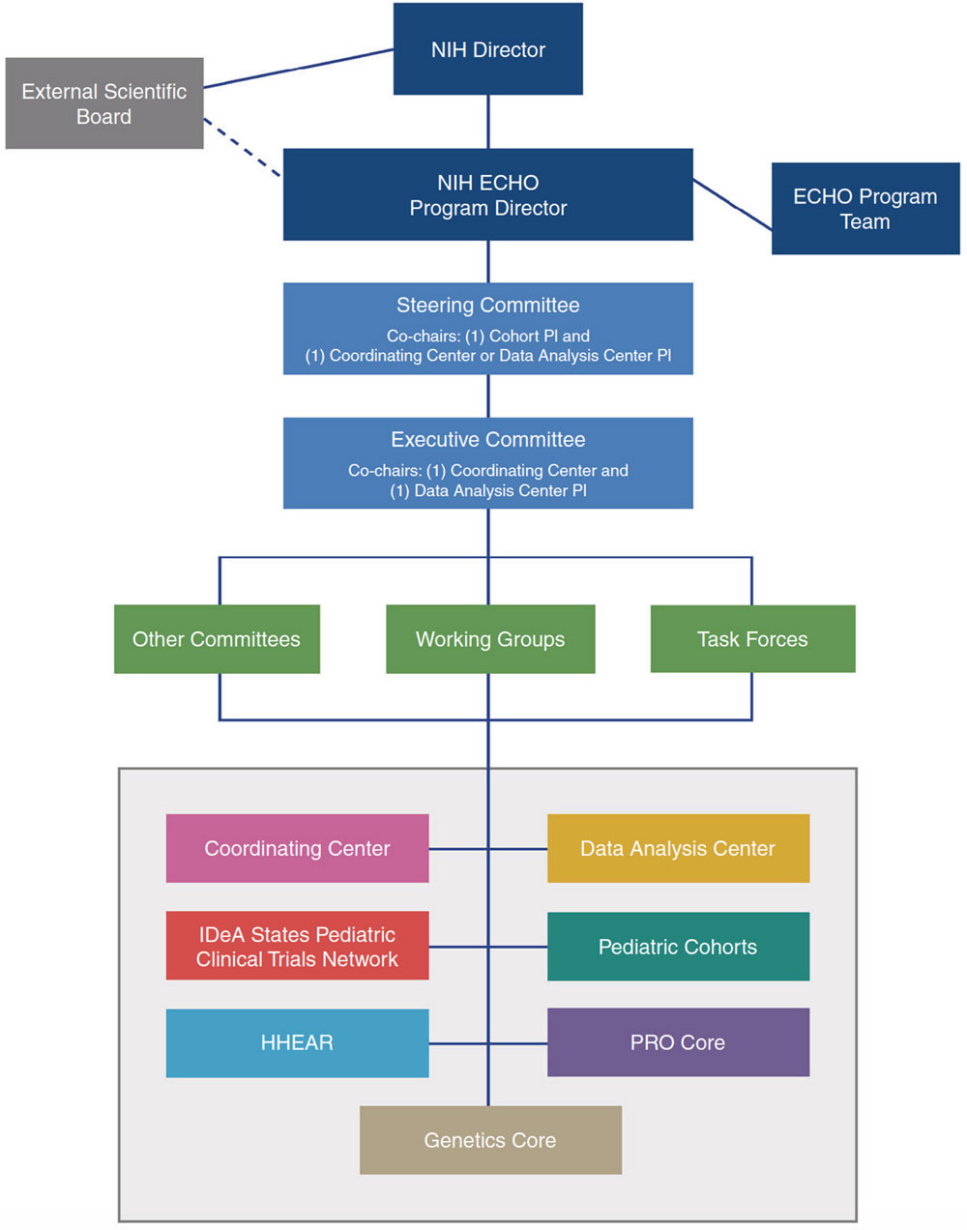
Organizational structure of the environmental influences on child health outcomes (ECHO) program. HHEAR = human health exposure analysis resource; IDeA =states institutional development award states; NIH = National Institutes of Health; PI = principal investigator; PRO = person-reported outcomes (from LeWinn et al. 2022 [40]).

In September 2016, the NIH launched the ECHO Program, a 7-year nationwide multisite collaborative research program encompassing both observation and interventional arms exploring the effects of a broad range of early environmental exposures on child health and development [15]. As of the seventh year of funding, ECHO has enrolled over 60,000 participants (from the prenatal period through adulthood) across 46 U.S. states and territories. ECHO comprises over 1,200 researchers across 84 initial observational cohorts, the NIH, the Coordinating Center (CC), Data Analysis Center (DAC), Human Health Exposure Analysis Resource (HHEAR), and Patient-Reported Outcomes Core (PRO Core) (Fig. 2). We did not include the interventional arm of ECHO, the IDeA States Pediatric Clinical Trials Network [16], as it does not utilize the same organizational structure and has different protocols and processes as a clinical trial arm. Finally, at the time of data collection, we could not identify representatives from the nascent Genetics Core for inclusion in the study.

### Study Population

We interviewed internal stakeholders from the ECHO components, seeking broad representation of research and administrative roles across ECHO [17–19]. All internal ECHO stakeholders were informed about the study via an internal email with study information and an interview invitation. Those interested contacted the research team via email to schedule an interview via Zoom. From the respondents, we purposefully selected a sample to ensure maximum variation that represented diverse perspectives (geography, role, gender, etc.) from groups that we hypothesized would have different experiences of ECHO [19]. We supplemented the initial invitation with targeted email invitations to gather additional perspectives (e.g., roles, ECHO components) not represented in the initial email volunteers. The Duke University IRB determined this study to be exempt.

### Data Collection

The research team developed a semi-structured interview guide informed by PRISM that explored topics identified in the literature and by stakeholders. Six members of the research team (EF, KS, GN, DN, CV, and KH), who had previously conducted qualitative research and were trained specifically on the PRISM-informed semi-structured interview guide by the lead author (EF), conducted 20–45-minute virtual interviews until thematic saturation was achieved [20,21]. The interviews were recorded, transcribed, and accessible only to research team members.

As quantitative implementation outcomes, we conducted secondary analysis of the Goals, Outcomes, Indicators, and Targets (GOITs), which are continuously collected, aggregated, and analyzed by the CC and the DAC. The ECHO GOITs are an evaluation plan developed to track progress along domains identified by ECHO-wide internal stakeholders as important to the success of the program. The metrics are organized around four goals: (1) enroll and retain a large and diverse group of participants in the ECHO-wide Cohort to answer key scientific questions; (2) collect and make high-quality data available for analysis; (3) collect, store, and use biospecimens and extant assay data to support ECHO-wide Cohort research; and (4) publish and disseminate high-quality, impactful science. Performance on these metrics is shared with internal stakeholders on a regular basis. The GOITs have evolved over time reflecting slight variations depending on the trajectory of the program overall.

### Data Analysis

Qualitative data were analyzed using template and matrix analysis methods to assess perceptions about ECHO implementation, document attitudes toward collaborative team science, and generate a formative understanding of multilevel contextual factors [22–24]. Using a template developed with deductive *a priori* PRISM domains, the analysis also captured inductive, emergent themes. Pairs of team members were assigned a subset of transcripts. To establish agreement and consistency, each transcript was summarized independently by two co-authors, and then the summaries were reconciled until consensus was reached. The summaries were next entered into a matrix (i.e., Excel spreadsheet) organized by PRISM domain (i.e., each tab was a separate PRISM domain), and each domain was analyzed vertically by individual team members through analytic memos, in which emergent themes were identified within each domain. The team collectively reviewed and discussed the analytic memos and identified the overarching themes that independently emerged across multiple PRISM domains.

We mapped the quantitative GOITs onto the RE-AIM framework to measure implementation outcomes (Table 1). We looked at *reach* as the number and representativeness of participants together with qualitative data on recruitment, especially focused on equity. We used *effectiveness* to understand broader outcomes, for which research publications were a proxy measure. Related qualitative data were interviewees’ perceptions of the potential impacts of ECHO science. We used our qualitative data to understand *adoption* through internal stakeholder perceptions and experiences. While much of the qualitative findings are related to *implementation*, we also mapped GOITs concerning data collection, harmonization, and analysis to understand program delivery outcomes [13]. We did not look at maintenance per se; ECHO is in the implementation phase. Once we completed analysis of the interviews, we integrated the qualitative and quantitative data using side-by-side comparisons in joint displays [11, 25].

**Table 1.** RE-AIM measures in ECHO (Year 6 GOITs)

RE-AIM measures	Measure concept	Measure definition/data collection (GOIT) by August 31, 2022
R – Reach	The absolute number and representativeness of individuals who are willing to participate in the Program	1. # of level 2 children enrolled 2. Race/ethnicity diversity of level 2 participants (pregnant women and child) across the ECHO-wide Cohort Qualitative data on recruitment and retention strategies that address equity
E – Effectiveness	The impact of the Program on important outcomes, including potential negative effects, quality of life, and economic outcomes. Heterogeneity of effects and reasons for success or lack of such	1. # of ECHO-wide Cohort proposals published from those proposed in Year 5 2. # of ECHO-wide Cohort presentations at scientific meetings Qualitative data on stakeholder perspectives on impact and outcomes
A – Adoption	Staff participation and stakeholder perceptions of adoption	Qualitative data collecting perspectives from internal stakeholders of ECHO program
I – Implementation	Stakeholders’ fidelity to the various elements of the Program’s protocol, including infrastructure, consistency, and time required	1. Number of biospecimen (non-DNA) samples from level 2 participants 2. % of completed HHEAR assay data made available by the DAC on the platform 3. % of extant assay data transferred from the Cohorts made available by the DAC on the platform 4. % of essential data elements for level 2 participants with at least 12 months in a life stage or who have already completed the life stage are on the platform Most of the qualitative data exploring perceptions of program implementation and its facilitators and barriers
M – Maintenance	The extent to which intervention is sustained	Does not apply at this stage

DAC = data analysis center; ECHO = environmental influences on child health outcomes; GOIT = goals, outcomes, indicators, and targets; HHEAR = Human Health Exposure Analysis Resource. Level 2 participants = ECHO Program participants consented to all elements of the study protocol.

## Results

We conducted 24 virtual interviews with ECHO stakeholders between February 2022 and May 2022 (Table 2). Most interviewees were affiliated with a cohort, which reflects the ratio of components of ECHO; we were able to gather the perspective of all the components except HHEAR and the NIH. NIH declined to participate and despite invitations directed specifically to HHEAR representatives, none volunteered to participate. Table 3 illustrates the emergent themes that each research team member identified in their thematic analysis when writing an analytic memo for that specific PRISM domain. The research team’s discussion of the thematic analysis identified three major cross-cutting themes that emerged independently across the PRISM domains (bolded in Table 3). We present below the findings in three overarching themes synthesized across PRISM domains: (1) collaboration and team science; (2) communication and decision making; (3) diversity, equity, and inclusion; and an additional theme (4) implementation outcomes, in which the qualitative interview and quantitative GOIT data are presented together by RE-AIM domains. Further representative quotations with the emergent themes in PRISM domains are included in Table 3 to illustrate the breadth of responses and perceptions about the implementation of ECHO, not all of which could be include in the narrative below.

**Table 2.** Interview participant characteristics

ECHO component	Cohort (15) Coordinating Center (CC) (4) Data Analysis Center (DAC) (3) Patient Reported Outcomes Core (PROCore) (2)
Role (in general terms)	Cohort investigator (3) Cohort study research staff (11) Cohort data manager (1) DAC administrator (1) DAC statistician/Data manager (2) CC administrator (1) CC program staff (3)
Location	Midwest (7) Northeast (5) Northwest (2) Southeast (6) Southwest (4)

**Table 3.** Representative quotations within the a priori PRISM domains

PRISM domain	Emergent themes	Representative quotations
Intervention *ECHO internal stakeholder perceptions of the intervention elements from the perspective of the organization and the participants*	Organizational Perspective – **Collaboration and Team Science** – **Communication** – **Leadership and Decision Making** – **Diversity, Equity, and Inclusion**	“[ECHO infrastructure] gives me a lot of different perspectives that I did not and would not have had otherwise because we have such a democratic way of doing things that can be frustrating because it has some inverse efficiency, but it certainly also gives a lot more detail and explanation as to why certain ways are being requested” (#1022)“in terms of ECHO supporting my role as a career, I feel like I'm getting leadership training, communication training, oral and written presentation skills, but also working together during team science to work on research questions that align with my particular research interests” (#1025)
	Participant Perspective – **Diversity, Equity, and Inclusion** – Recruitment and Retention* – Participant Burden	“these are underserved, historically under represented community populations, and when I look around at who the PIs are they all have my skin color (white) so I don't like it, I think it’s a problem, but… who am I?” (#1008)
Recipients *The organization and participant characteristics* *which influence the Program’s ability to be implemented*	Organizational Characteristics – **Collaboration and Team Science** – **Communication** – Resources (Staffing)	“I think that the cohorts that don't go by a medical model are sometimes forgotten about…So adoptive parents in our sample are considered caregivers under ECHO whenever data is imported they're not even considered parents, which is really like awful. But the same thing about step-parents who may be the person that’s primarily raising the child and they're not considered parents, because they're not birth parents.” (#1014)“I think that’s one of the problems of our cohort is that they are too homogeneous. I think if we reach out to the Community, maybe we can diversify a little bit” (#1007)
	Participant Characteristics ECHO participant demographics*	“You know some of the folks in our community work 12-hour shifts and then they go home and work their second shift with their families, because they live in a communal household. They don't get an hour to sit, they just don't get an hour and that’s just the reality of it so nope to those questionnaires. You aren't going to get them and it’s probably not that they don't want to do them, they just can't.” (#1009)
Implementation infrastructure *The infrastructure within a given context which influences implementation and sustainability of the Program*	– **Collaboration and Team Science** – **Communication and Decision Making** – Leadership – Resources (Staffing)	“We have a good structure, but it often feels like we're always kind of chasing to catch up, especially when it comes to data. The DAC is doing amazing. I don't know even how they're doing it. I don't know if more people would help though in terms of speeding up the de-identification of data or doing analyses.” (#1020)“What I would be looking for, and this is probably different than anybody else, is some stronger federalism. Let’s be starfleet on this and see how we can better work together.” (#1003)
External environment *Elements such as payors, policy, or competition which influence implementation*	– Funding – COVID-19 pandemic – Big wins* ○ Population benefits ○ Scientific community ○ Research methods	“I've been surprised at how hard remote data collection has been. I've been surprised at how difficult the concept that I thought was going to be an easy win was. We’re in COVID, it’s a once in a lifetime situation. We know you want to pitch in and help us learn more about COVID. I’m sure that message resonated but at the same time, people were just too overwhelmed.” (#1010)“we have communicated with our participants, so they are aware of what ECHO is when we do send out our newsletters and infographics you know they are aware that they are part of something bigger or that they can take part in something much bigger” (#1012)

PRISM = practical, robust implementation and sustainability model [8]; CC = coordinating center; DAC = data analysis center; ECHO = environmental influences on child health outcomes; GOITs = goals, outcomes, indicators, and targets.

**Bolded** emergent themes signify overarching themes elaborated in narrative text, emergent themes with an (*) were aligned with RE-AIM implementation outcomes.

### Collaboration and Team Science

The benefit of collaboration and team science, broadly defined as experiences with other internal elements of ECHO, was the most common cross-cutting theme that emerged across the Intervention, Recipient, and Implementation Infrastructure domains. Many interviewees enjoyed being part of a national study that brings greater breadth and depth of expertise to the team and more exciting translational work and clinical research. One respondent explained:

People have begun to find a lot of commonalities across not just the outcomes, but the exposures and finding different and new ways to collaborate with each other. I’m writing a paper right now that includes 47 cohorts, and so we have 40 plus co-authors and that’s huge (#1020).

Many felt that ECHO had enhanced the science of their specific cohort with its expanded focus aligned across multiple exposures and outcomes. However, some interviewees who felt they had willingly adapted their original study plans to meet overall ECHO goals were frustrated that some ECHO colleagues did not prioritize collaboration over their own research. Nonetheless, interviewees enjoyed opportunities to collaborate, both at large in-person and smaller group virtual meetings, which were seen as opportunities to learn from their ECHO colleagues. Many noted that while the first few years of the program felt chaotic, things had progressively stabilized and become more standardized and streamlined with greater focus and guidance.

Most expressed a desire for more opportunities for direct connection, learning, and sharing. Interviewees wanted more time to “build camaraderie within so that we could learn from each other” (#1009). Respondents felt there were opportunities for cohorts to support each other, acknowledging that many of the barriers were common, such as aligning the needs of similar groups of participants throughout the cohorts and leveraging the expertise of those working out in the field. There were requests for opportunities for problem solving and collaboration at the staff research level:

…more communication between cohort study coordinators, I just personally think would be super helpful. When we used to go like in person to our in-person meetings in DC or like the larger ECHO-wide meetings pre-COVID. The most I got from that was a breakout groups talking other coordinators, just like hearing their opinions and talking through like different scenarios and issues with them, I think, was like the most helpful out of those meetings. (#1001).

Another interviewee shared:

I know there’s like the places on the [online portal] where people can share these resources, but they're not utilized. I do not have an answer for how, but I just I know that collectively we have resources and strategies that would be beneficial to each other (#1014).

The interviewees spoke positively about the program’s collaboration: “ECHO has proven that you can have all these various cohorts and all these various components within a program work and be successful” (#1004), and, “I'm definitely learning… how to work with all these different cohorts that have different data structures and study structures and it’s been really interesting to kind of learn the sides of the science and contribute to that part of it” (#1018).

### Communication and Decision Making

The theme of communication and decision making also emerged across the Intervention, Recipient, and Implementation Infrastructure domains. This theme encompassed responsiveness between ECHO components, internal meetings, requests, and infrastructure elements such as data harmonization. While those interviewed generally felt that ECHO is well organized, some reported varied experiences and impacts on their work. Communication among the ECHO components was characterized as initially complicated – one interviewee said ECHO, “needs better communication and a better decision making process (#1009)” – but progressively getting better. Multiple respondents perceived a long time to get answers to routine requests from the DAC or CC, which was said to delay staff entry into the field or create inefficiencies when data collection continues occurring in real time:

We might be trying to do something like a sample collection, and we need to know something specific and it takes a while to…find out the answer, and then in that meantime you've…maybe not been collecting the sample…now you’re behind (#1002).

At the same time, some interviewees expressed difficulty with receiving multiple requests at the same time, all with seemingly urgent deadlines. Respondents sometimes felt:

there’s a big disconnect maybe even just in the urgency of it. A lot of times we're given a week or two maybe to respond… month or two would be more realistic fitting in with everything else that teams are doing (#1017).

However, interviewees recognized that the size of ECHO impeded timely, clear, and efficient communication:

I think that that’s possibly just a function of the size of ECHO and how many, you know how many people need to be behind each decision and things like that it might not be easy to always give a quick answer (#1002).

Respondents identified the opportunity for greater transparency in decision making, like clarifying who in the organizational chart answers which questions when and whose needs are prioritized:

It seems like in ECHO everything has to go to committee, everything has to be discussed and checked with everyone else and there’s not a sense of collective trust like you know I’m going to just let somebody else make that decision and not worry about it… let’s move on it’s just there’s a lot of checking and rechecking and you know. People are spending a lot of time in meetings that you know, sometimes an hour will go by and I will ask myself what really got accomplished there? (#1021).

The size and complexity of the consortium added to the greater need for transparency considering the understanding that everyone has their own perspective, which can impede communication. One ECHO Program member commented, “It feels like sometimes you talk to people and they feel like their one small thing is the most important thing, but it’s like you’ve got to remember that everyone has their small thing*”* (#1018).

Descriptions of the development of the ECHO-wide data collection protocol and the current data processes (e.g., collection, cleaning, harmonization, etc.) reflected some of the challenges and opportunities in decision making. Respondents discussed how more work should have been done up front to streamline the protocol and expressed hope that it would be more aligned in the future:

It’s been a challenge in terms of just kind of trying to be both flexible to respect where the cohorts came in, but also have something standard so that we can create this database…I think that was one and still is one of the biggest challenges of having everyone participate in a standard protocol and be able to have usable data (#1020).

Another interviewee added:

I think we needed to put more time in up front on definition and harmonization activities and not do it at the backend. I think that will be our limitation throughout the rest of the current years and I think it will haunt us in the next phase a little bit as well. So, I think that if I had to do some things over again, that would be something that I think we should have put more energy in up front and defined things much better (#1006).

Interviewees mentioned the perceived burden on ECHO participants of the Program protocol as another opportunity for further alignment and greater flexibility. The sheer number of elements were often cited as a concern for families, “because the protocol is so much more extensive and they’re targeting multiple people in the same household, so the contact is more frequent, it has been hurting retention” (#1015). Respondents generally felt that streamlined decision making would help focus the protocol.

### Diversity, Equity, and Inclusion (DEI)

Diversity, equity, and inclusion (DEI) emerged across multiple PRISM domains both explicitly (e.g., Intervention) and implicitly, in relation to implementation outcomes such as recruitment and retention. Although *diversity* was not specifically defined in the interview script, interviewees tended to interpret this term as being in reference to underrepresented or non-White groups. Interviewees recognized that DEI had been well prioritized. “DEI was obviously going on in our home base, but ECHO has really educated me a lot about it and given the opportunities for trying to advance that field” (#1010). ECHO’s focus on DEI some years after the start of the program left some cohorts feeling they couldn't respond as well because they could only recruit from the participants of their original study. Conversely, the ECHO cohorts that were still recruiting were happy to have the chance to meet the DEI-related goals. Other cohorts felt that access to more diverse families was lost both in the longer time it took to start implementing the protocol. However, most of the cohorts had recruited underserved populations initially, so interviewees felt that DEI was nevertheless supported and maintained.

In addition to ECHO participation, interviewees described different avenues to enhance and ensure DEI across ECHO. One interviewee described their cohort’s efforts to ensure biospecimens are representative of all children in their study sample:

For example, we have hair collection videos so sort of like how to collect your own hair and we've recently added type 5 hair, African American, different types of hairstyles, we've created 4 different additional videos. So that people with braids or locks can have a video that’s more tailored to them, instead of watching somebody else with type 4 hair but loose (#1014).

### Implementation Outcomes

Our data on implementation outcomes are both qualitative and quantitative; the interview themes are presented alongside the GOITs that have been mapped onto the RE-AIM measures (Table 1). The GOITs from our data collection period provide context for the interviewees’ experiences and perceptions. They also provide insight into the shared priorities and how the program itself assesses implementation. The GOITs were also a specific domain of interest in the interviews, so we present our qualitative findings about the GOITs to frame the results in the implementation domains.

### Goals, Objectives, Indicators, and Targets (GOITs)

We present data from the GOITs for Year 6 (2021–2022) collected concurrently with the semi-structured interviews (February–May 2022). The GOITs were developed initially in the fifth year of ECHO by a task force of internal stakeholders, based on expert recommendations for evaluation and program-specific metadata, such as number of cohorts, proposals in the pipeline, etc.; the objectives are adjusted annually. For example, the task force initially identified the dissemination targets (60 publications and presentations per year) based on the number of cohorts in ECHO, and then used metadata from the publications pipeline to predict that target in future iterations. Similar to other ECHO infrastructure, interviewees felt the process of identifying and selecting GOITs improved over time:

I think they're somewhat useful, probably not as useful as the amount of time we've dedicated to talking about them and developing them and studying them and figuring out how to measure them… I think it is important to have these targets because of our competing priorities, but we seem like we spent a lot of time talking about that that could have been spent on other things, like writing papers (#1010).

In general, they felt the metrics are important:

I think they're really helpful to have metrics that you know you're being measured by rather than just you know, trying to do everything right but not sure exactly what is the most important. So, I think it’s really helpful to have written metrics that everyone’s held to the same metrics (#1002).

While respondents felt the GOITs were a useful framework for articulating, monitoring, and focusing attention on program priorities—important given ECHO’s scientific and operational complexity—there were concerns about feasibility. Some interviewees expressed concern about achieving targets for enrollment, retention, and data collection that were impacted by things outside of their control (e.g., COVID, rurality). One interviewee explained that GOITs are “useful in knowing what the larger ECHO program is looking for, but I do not think it represents completely the work that’s being done at the local sites*”* (#1017). Others expressed additional concerns, “data collection goals are harder for rural sites relying on remote data collection,” (#1012) and “I think sometimes depending on the goal, it could take away from some of the science. If we say we want X number of publications--that’s great and that would be wonderful--but we do not want just to churn out publications for publication’s sake, because science can take a while” (#1020).

### Reach

We defined the reach measure concept as the absolute number, proportion, and representativeness of individuals who are willing to participate in the ECHO Program, with a focus on recruitment strategies. The reach domain aligned with ECHO GOIT A.1: Race/Ethnicity of Pregnant Women and Child Participants Across the ECHO-Wide Cohort. At the time of the interviews, the ECHO-wide program was at or very close to the targets set for these metrics (Table 4). The related qualitative findings from the interviews addressed opportunities to enhance strategies for more diversity in recruitment and retention.

**Table 4.** Goal A: enrollment and retention of participants

A1 Objective: Enroll historically underrepresented participants				
Indicator: Race/Ethnicity diversity of pregnant women and child participants across ECHO				
Target: Race≥55% other than non-Hispanic White; Ethnicity ≥ 25% Hispanic				
Month-year	Non-White pregnant women (%)	Non-White child participants (%)	Hispanic pregnant women participants (%)	Hispanic children (%)
Feb-22	54	55	24	23
Mar-22	54	55	24	24
Apr-22	55	55	25	24
May-22	55	56	25	24
Jun-22	54	56	25	24

A2 Objective: Meet projection for enrollment of children and pregnant women		
Indicator: Number of children enrolled		
Target: 43,500 children enrolled		
Month-year	Children enrolled (*N*)	Percent of target
Feb-22	29,354	67
Mar-22	30,058	69
Apr-22	30,467	70
May-22	30,858	71
Jun-22	31,283	72

ECHO = environmental influences on child health outcomes; N = number.

Several interviewees expressed the need for more cultural competency in ECHO, especially in terms of the marked differences in how particular groups should be approached:

People don't understand what it means to study Dominicans, like Latinx Dominicans are very different from Mexicans, who are very different from Puerto Ricans and so I think that there just isn't enough attention to cultural detail and cultural competency in ECHO at all (#1008).

This case was also made in reference to enrolling and retaining indigenous families, which also requires acknowledgement of historical injustices, attention to trust building, and a greater investment in resources:

Those communities take more time, they just take more time… You don't start off with the questions right away, you see how their family is doing, and how their kids are doing, and you go back years first, before you get to the first question… with the American Indian population that we work with it’s all relationships, it’s all trust, it’s all that that’s what that culture, you know really thrives on… it’s all about relationship building (#1009).

Resources and time were cited as critical both to reaching more underserved or minoritized communities and keeping them engaged in research.

### Effectiveness

Effectiveness is the impact of the program on important outcomes, for which research publications using ECHO-wide data (versus single cohort studies) were a proxy measure. Table 5 shows data on (1) the number of ECHO-wide Cohort manuscripts published that were derived from proposals submitted during the previous grant year, and (2) the number of ECHO-wide Cohort presentations at scientific meetings. By the end of the data collection period, the program was not on target to reach its goals by the end of the year, likely due to delays in initial infrastructure (e.g., data harmonization) building. However, the ECHO Publications Committee received a 44% increase in manuscript submissions and an 867% increase in presentation submissions compared to those received during grant year 5. Our qualitative data focused on the broad impacts for the public and the scientific and research communities.

**Table 5.** Goal D: publication and dissemination

D1.1 Objective: Publish ECHO-wide cohort analyses from year 5			
Indicator: # of ECHO-wide cohort data analyses published from those proposed in year 5			
Target: 60 (annually)			
Month-year	Publications (*N*)	Percent of Target	Cumulative
Sept-21–Feb-22	4	7	4
Mar-22	1	8	5
Apr-22	1	10	6
May-22	0	10	6
Jun-22	3	15	9

D3.1 Objective: Disseminate results to key stakeholder organizations			
Indicator: # of ECHO-wide cohort presentations at scientific meetings			
Target: 60 (annually)			
Month-year	Presentations (*N*)	Percent of target	Cumulative
Sept-21–Feb-22	8	13	8
Mar-22	1	15	9
Apr-22	6	25	15
May-22	4	32	19
Jun-22	10	48	29

ECHO = environmental influences of child health outcomes; N = number.

Most interviewees mentioned the broad hope that ECHO would result in healthier children and would benefit ECHO participants, families, and the public at large through better understanding the impact of exposures on child health. Interviewees cited the healthcare system, economic system, and entire country, while others described potential benefits focused on specific groups (e.g., children with a specific diagnosis). Several interviewees mentioned ECHO’s potential impact at the policy level:

It’s going to take some time, for these analysis and data cleaning but I’m hoping it turns into some type of public policy where they're able to see that this geographic region of the US had a higher incidence of you know, this type of marker which we then were able to link to this, you know household chemical you know, maybe it will impact policy in that manner, or at least put out warnings for people” (#1012).

Many interviewees described benefits to the scientific and research community, including best practices and lessons learned for team science, large-scale studies, and collaboration. Several interviewees mentioned how it would benefit individual researchers’ careers, especially junior investigators, in terms of experience and networking. “It will help people’s careers… it’s going to, you know, find whoever the next director of NIEHS is, or the NICHD” (#1008).

A few interviewees mentioned specific outcomes, exposures, or health conditions that ECHO research could impact significantly, including Attention-deficit/hyperactivity disorder (ADHD), autism-spectrum condition (ASC), and asthma:

The biggest ones that I think will actually happen are probably going to be something about asthma, because we'll have a lot of sample size and, maybe, be able to find some environmental relationship that could lead to actual regulation (#1003).

ECHO research in genetics, genomics, and epigenomics was described as, “poised to make some true discoveries on health outcomes and maybe even therapeutics” (#1006), and “chemical exposure data from biospecimens we can assay that will give us rich exposure data from biospecimens that we can link to later child health outcomes” (#1025).

Several interviewees mentioned new research methods developed over the course of the program which would benefit the scientific community for years to come, including new measures and methods. As a result of ECHO, the Patient-Reported Outcomes Measurement Information System (PROMIS) team has developed new measures and validated others for younger ages, expanding our ability to understand impacts at earlier life stages [26]. The COVID-19 pandemic also prompted advances in remote data collection:

Information gained during the pandemic can help researchers move more to remote. I think we can get harder-to-reach populations, even outside of the pandemic, I think we can get harder to reach populations if we have better recommendations for how to collect all sorts of data remotely, and I think that that can be gained from this, because we're collecting so many different types of data (#1014).

Some also saw benefits in the ECHO dataset itself as a long-term scientific resource; the sheer size and diversity of the sample, especially populations that have previously been excluded from research (e.g., Indigenous populations) [27]. Interviewees described opportunities arising from the ECHO data infrastructure including the harmonization of the extant data, “harnessing past existing data to lead to new research questions*”* (#1013), the opportunities for intergenerational studies, and the ability to look at longitudinal data together with biospecimens. Going forward, deidentified data is available for investigators for scientific purposes by applying to the Eunice Kennedy Shriver National Institute of Child Health and Human Development (NICHD) Data and Specimen Hub (DASH) [28].

### Adoption

We considered adoption in terms of both ECHO Program participants and ECHO internal stakeholders. With regards to the former, interviewees had considerable variation in their observations of participant experience; they were both encouraging and challenging. One cohort described having very committed families and gave the example of a dad continuing to participate when the mom passed. ECHO participants were described as engaged, with low attrition, but even when recruitment and retention was good, there remained the problem of getting participants to commit to the study visit schedule.

For internal stakeholder adoption, respondents mostly spoke positively about the investigative network and opportunity to do collaborative science, although some felt that there was not enough inclusion of team members working across multiple roles*: “*I have a doctorate and…I have been involved on one of the writing teams, but I think it’s very cliquey*”* (#1008). Interviewees shared great advantages to participating in a large research consortium, including many stakeholders willing to listen to ideas:

“I'm definitely learning…how to work with all these different cohorts that have different data structures and study structures and it’s been really interesting to kind of learn the sides of the science and contribute to that part of it*”* (#1018).

However, respondents noted inefficiencies such as investigators sometimes having to do things outside their expertise to meet Program goals. One interviewee offered their perspective on the reason for challenges with alignment:

We task a lot of investigators with doing things in ECHO that is not in their area of expertise. We do it for the inclusiveness and to make sure voices are heard, but… They're not getting paid to figure out how to track publications or even things around biospecimens… But in ECHO, we take a lot of that responsibility, hand it off to a committee who doesn't really have to sweat about it, because at the end of the day, if it doesn't get done, they weren't getting paid to do it… That committee comes up with something and we roll it out and still half of everybody’s mad. It’s that balance of being able to listen to voices, but get things done (#1019).

### Implementation

We considered implementation in terms of internal stakeholders’ fidelity to elements of ECHO, including data collection and analysis infrastructure. The GOITs that aligned with this concept concerned data, biospecimens, assays, and completeness of data collection to support program-wide research (Table 6). During our study, ECHO was meeting some goals while there was less progress towards others. The qualitative data reflected challenges with alignment and opportunities for enhanced collaboration.

**Table 6.** Goal C: biospecimens and assays

C1.1 Objective: Deposit essential biospecimens		
C1.2 Indicator: # of non-DNA samples from participants in the biorepository		
C1.2 Target: 70,000 non-DNA samples from participants in the biorepository		
Month-year	Participants' non-DNA biospecimens (*N*)	Percent of target
Sept-21–Feb-22	27,151	39
Mar-22	31,744	45
Apr-22	34,161	49
May-22	36,766	53
Jun-22	38,246	55

C3.3 Objective: Make biospecimen assay results available on the ECHO-wide data platform			
C3.2 Indicator: % of completed HHEAR assay data made available by DAC on the platform within 30 days of receipt			
C3.2 Target: 100% of completed HHEAR assay data made available by DAC on the platform within 30 days of receipt			
Month-year	Completed HHEAR assay data transferred to DAC (*N*)	Completed HHEAR assay data available on platform within 30 days of receipt (*N*)	Percent of completed HHEAR assay data made available on platform within 30 days of receipt
Sept-21–Feb-22	2	1	50
Mar-22	0	0	NA
Apr-22	0	0	NA
May-22	2	0	0
Jun-22	0	0	NA

C3.1 Objective: Make biospecimen assay results available on the ECHO-wide data platform			
C3.3 Indicator: % of extant assay data transferred from the cohorts made available by the DAC on the platform within 60 days of transfer			
C3.3 Target: 100% of extant assay data transferred from the cohorts made available by the DAC on the platform within 60 days of transfer			
Month-year	Cohorts that transferred extant assay data (*N*)	Cohort’s extant assay data available within 60 days (*N*)	Percent of extant assay data available within 60 days of transfer
Sept-21–Feb-22	13	3	23
Mar-22	5	5	100
Apr-22	2	2	100
May-22	1	1	100
Jun-22	1	0	0

DAC = data analysis center; ECHO = environmental influences on child health outcomes; GOIT = goals, outcomes, indicators, and targets; HHEAR = human health exposure analysis resource; N = number.

Multiple respondents cited challenges around flexibility with data submission and extant data harmonization. Some interviewees expressed difficulty managing diverse stakeholders with different research priorities:

Anything that we do to promote uniformity makes cohorts less happy. We have to have the cohorts, we have to have people engaged with particular participants, and wanting to do the ECHO protocol, but if everybody’s going in 70 directions, we generally do not get anything done (#1019).

However, most interviewees felt that ECHO-wide sharing and data management infrastructure were relatively well-organized and helpful, but that getting new people access and trained in the systems was often difficult given the turnover in such a long project, “I mean it takes six months to get a research assistant trained up. I mean, yes, you’re behind the curve when they start” (#1009).

## Discussion

Our findings provide insights into ECHO’s internal stakeholders’ perceptions about the contextual factors impacting program implementation. While (1) collaboration and team science, (2) communication and decision making, and (3) DEI were major cross-cutting themes, there was considerable heterogeneity among interviewees’ perceptions and descriptions of facilitators and barriers to the implementation of ECHO. Whether certain factors were considered to facilitate or hinder program success reflected the variation in perspectives, training, roles, etc. across the program. This diversity of experiences illustrates that ”best practices” for large research consortia may not be one-size fits all, and implementation and engagement likely need to be tailored for different groups, both internal and external stakeholders. While overall committed to and excited by the opportunities afforded by multisite collaboration, the size and complexity of the program sometimes left individuals feeling frustrated or adrift.

These findings may reflect the dearth of published implementation studies about large multidisciplinary research consortia. The literature so far has centered on consortia focused on a specific disease state (e.g., human immunodeficiency virus (HIV)), which would allow for more immediate alignment of goals and procedures, or supporting collaboration across smaller projects, for which the issues are different [29–31]. Additionally, previous research to understand team science in the context of large research consortia has employed exclusively quantitative methods (e.g., surveys and social network analysis) [7,8,32–36].

Our study contributes a theory-informed mixed methods approach to understanding implementation of a large, multidisciplinary research consortium. Our findings also offer an in-depth understanding of why and how stakeholders collaborate, and what works to produce impactful science. Previous analysis of research stakeholder engagement has offered a classification of four types of impact: (1) conceptual (changing knowledge, understanding, and attitudes); (2) instrumental (changing policy and practice, given research findings); (3) capacity-building (changing researchers’ ability to conduct future work), and (4) connectivity (shaping the existence and strengths of networks of people and organizations using the research) [37,38]. In this context, our qualitative and quantitative data provide insight into how the infrastructure and contextual factors of a large longitudinal research consortium produces impact for its stakeholders. The overarching theme from respondents was that team science, co-learning, and collaboration were the most valued and important elements; they saw opportunities and lessons learned around ways to enhance communication and collaboration. Additionally, inclusivity for both internal ECHO stakeholders (across components, roles, etc.) and external stakeholders (especially engagement with underrepresented and historically marginalized communities) was commonly identified as an overarching, guiding principle going forward.

### Limitations

It is possible that the interviewees were not representative of all ECHO stakeholders, but our purposeful and targeted sample attempted representation as broad as possible. One major limitation is that we were not able to include ECHO participants in the interviews, which would have provided an important perspective, especially with regard to the Recipient PRISM domain. Future implementation studies should include participant voices and selected representation from every ECHO component (i.e., HHEAR) as well as other stakeholder groups external to ECHO to make the picture more complete. Ongoing work is assessing participant experience and perceptions of burden directly from the participants themselves in a participant feedback instrument that is part of the ECHO protocol. This will provide valuable insight into how elements of participant experience (time spent, participation valued, satisfaction with level of return of results, the role of compensation and duration of study involvement) vary by participant characteristics and interactions with the ECHO study. Additionally, although the Year 6 GOITs may not have been the most accurate way to measure and evaluate ECHO Program implementation quantitatively, we explored respondents’ overall perceptions of GOITs in general as a useful resource for program implementation during the interviews. Future research should include considerations of maintenance (e.g., the “M” in RE-AIM), especially considering how crucial ongoing participant engagement is for long-term observational studies.

This research may be analytically generalizable and transferable to other large research consortia, and could benefit a wide range of stakeholders, including funding organizations [39]. The implementation science structure organizes mixed methods data collection and analysis to provide a real-time understanding of implementation to ensure impactful science. Finally, our interviewees’ perspectives provide investigators and researchers with insights into participation in large transdisciplinary research consortia.
